# Adoptive cellular therapy using bone marrow-derived T cells for the treatment of childhood leukemia

**DOI:** 10.1186/2051-1426-3-S2-P15

**Published:** 2015-11-04

**Authors:** Jill Gershan, Katie Palen, James Weber, Monica Thakar, Bryon Johnson

**Affiliations:** 1 Medical College of Wisconsin, Milwaukee, WI, USA

## Background

The survival for pediatric leukemia patients is approaching 90%, but there are patient subgroups that fare poorly including those with relapsed, or high-risk, acute lymphocytic and acute myeloid leukemia (ALL and AML, respectively). For these reasons, there is an unprecedented need for more effective therapies. Adoptive cellular therapy (ACT) has been shown to be an effective anti-tumor immune therapy in some patients.

## Methods

We are assessing the feasibility and potential anti-cancer benefits of using BM-derived T cells for ACT treatment of childhood leukemia. Leukemic cells accumulate in the bone marrow (BM) and the BM is a rich source of antigen-experienced memory T cells. Our goal is to optimize *ex vivo* expansion of BM-derived T cells in order to produce a pool of leukemia-specific T cells with anti-tumor memory. For murine studies, BM-derived T cells harvested from naïve mice or mice harboring C1498 AML are expanded *ex vivo* in a mixture of common-gamma chain cytokines (IL-2, IL-7, IL-15 and IL-21). Prior to and following expansion, T cells are analyzed for expression of phenotypic markers, fold-expansion, apoptosis, and the spare respiratory reserve (SRC, as a general measure of mitochondrial function).

## Results

To date, we have determined that murine T cells optimally expand in IL-2, IL-7 and IL-15 with less apoptosis than when expanded in IL-2 alone, IL-2, IL-7 and IL-21 or IL-2, IL-15 and IL-21. Murine BM-derived T cells have greater SRC and are less apoptotic than T cells derived from the spleen. The *in vivo* persistence and anti-leukemia efficacy will be determined after expanded congenic T cells are delivered as ACT to C1498 bearing mice. For human studies, peripheral blood and bone marrow is being collected from pediatric patients with ALL or AML. The T cells are expanded in IL-2, IL-7 and IL-15 and analyzed similar to the murine T cells. Consistent with the murine data, human peripheral blood and BM-derived T cells expand efficiently in IL-2, IL-7 and IL-15. BM-derived T cells are less apoptotic than T cells expanded from the periphery (blood).

## Conclusions

These studies will provide a foundation for the development of a clinical ACT protocol using BM-derived T cells for the treatment of childhood leukemia.

